# Human Articular Chondrocytes Retain Their Phenotype in Sustained Hypoxia While Normoxia Promotes Their Immunomodulatory Potential

**DOI:** 10.1177/1947603518769714

**Published:** 2018-04-19

**Authors:** Claire Mennan, John Garcia, Helen McCarthy, Sharon Owen, Jade Perry, Karina Wright, Robin Banerjee, James B. Richardson, Sally Roberts

**Affiliations:** 1 The Robert Jones and Agnes Hunt Orthopaedic Hospital NHS Foundation Trust, Oswestry, Shropshire, UK; 2 Institute for Science & Technology in Medicine, Keele University, Keele, Staffordshire, UK

**Keywords:** Sustained hypoxia, hypoxic workstation, chondrogenic, immunomodulation, cartilage repair

## Abstract

**Objective:**

To assess the phenotype of human articular chondrocytes cultured in normoxia (21% O_2_) or continuous hypoxia (2% O_2_).

**Design:**

Chondrocytes were extracted from patients undergoing total knee replacement (*n* = 5) and cultured in ~21% (normoxic chondrocytes, NC) and 2% (hypoxic chondrocytes, HC) oxygen in both monolayer and 3-dimensional (3D) pellet culture and compared with freshly isolated chondrocytes (FC). Cells were assessed by flow cytometry for markers indicative of mesenchymal stromal cells (MSCs), chondrogenic-potency and dedifferentiation. Chondrogenic potency and immunomodulatory gene expression was assessed in NC and HC by reverse transcription quantitative polymerase chain reaction. Immunohistochemistry was used to assess collagen II production following 3D pellet culture.

**Results:**

NC were positive (>97%, *n* = 5) for MSC markers, CD73, CD90, and CD105, while HC demonstrated <90% positivity (*n* = 4) and FC (*n* = 5) less again (CD73 and CD90 <20%; CD105 <40%). The markers CD166 and CD151, indicative of chondrogenic de-differentiation, were significantly higher on NC compared with HC and lowest on FC. NC also produced the highest levels of CD106 and showed the greatest levels of *IDO* expression, following interferon-γ stimulation, indicating immunomodulatory potential. NC produced the highest levels of CD49c (>60%) compared with HC and FC in which production was <2%. Hypoxic conditions upregulated expression of *SOX9*, frizzled-related protein (*FRZB*), fibroblast growth factor receptor 3 (*FGFR3*), and collagen type II (*COL2A1*) and downregulated activin receptor-like kinase 1 (*ALK1*) in 3 out of 4 patients compared with normoxic conditions for monolayer cells.

**Conclusions:**

Hypoxic conditions encourage retention of a chondrogenic phenotype with some immunomodulatory potential, whereas normoxia promotes dedifferentiation of chondrocytes toward an MSC phenotype with loss of chondrogenic potency but enhanced immunomodulatory capacity.

## Introduction

Autologous chondrocyte implantation (ACI) usually involves cell expansion in normoxic conditions (21% O_2_) *in vitro*, although, chondrocytes are adapted to metabolize at much lower O_2_ concentrations *in vivo*.^1^ Early studies suggested that O_2_ tension may be as low as 1% to 2.5% in the mid-zone of articular cartilage^2^ and even lower in the deep zone. Chondrocytes cultured in monolayer under normoxic conditions have been shown to dedifferentiate to a more fibroblast-like phenotype,^3,4^ which is thought to result in the formation of a fibrocartilaginous repair tissue *in vivo*, that is biomechanically inferior to hyaline articular cartilage produced by chondrocytes.^5^ Hypoxic culture conditions could help maintain the chondrocyte phenotype and prevent dedifferentiation.^6^ Characterization and assessment of the dedifferentiation of chondrocytes, as well as the chondrogenic potential of mesenchymal stromal cells (MSCs), has mainly relied on the expression of *SOX9* and the production of type II collagen and proteoglycan. However, little is known about the changes in the cell surface marker expression during dedifferentiation. For example, according to the International Society for Cell Therapy (ISCT) criteria for defining MSCs, cells should be positive for CD44, CD90, CD73, and CD105 and negative for hematopoietic markers such as CD14, CD34, CD45, and HLA-DR. However, this pattern of production has been shown to change depending on the culture conditions or cell stimulation on MSCs,^7,8^ making comparisons difficult between studies. Some studies have clearly demonstrated that monolayer expansion of chondrocytes in normoxia induces changes in the expression of several surface markers and that the profile of dedifferentiated chondrocytes has a striking resemblance to that of MSCs.^9^ Several cell surface markers such as CD151, CD166, and CD44 have been proposed as being indicative of chondrogenic potency on both MSCs and chondrocytes,^10-13^ but the expression profiles of these markers in normoxic and hypoxic conditions have not yet been thoroughly investigated. Reports on the cell surface marker profile of chondrocytes maintained in hypoxia often relate to cells grown in intermittent hypoxia (e.g., when cells are returned to 21% O_2_ for feeding and passaging or media is used that is not conditioned to low oxygen).^14^

MSCs used in cell therapies are increasingly thought to exhibit their therapeutic effect via a paracrine mechanism,^15,16^ producing immunomodulatory and anti-inflammatory molecules. One of the most potent immunomodulatory molecules produced by MSCs, when primed with inflammatory cytokines, is indoleamine 2, 3-dioxygenase (IDO), an enzyme that regulates T-cell proliferation.^17^ Chondrocytes expanded in monolayer in normoxia, which exhibit cell surface markers and morphologies similar to MSCs, may also produce immunomodulatory proteins.

Together with the fact that there is little information on the cell surface marker profile of chondrocytes maintained in sustained (not intermittent) hypoxia, we designed a study to assess the extent of dedifferentiation and the associated phenotypic and cell surface marker expression on chondrocytes in culture expanded continuous hypoxia. We have studied the *in vitro* growth of chondrocytes in 2% and 21% O_2_, with the long-term aim of understanding the most likely mechanism of action (either paracrine or formation of repair tissue) of these cells on return to the patient to determine if hypoxic culture conditions may provide a better growth environment for cells destined for cartilage repair.

## Methods

### Chondrocyte Isolation and Culture

**Figure 1.**
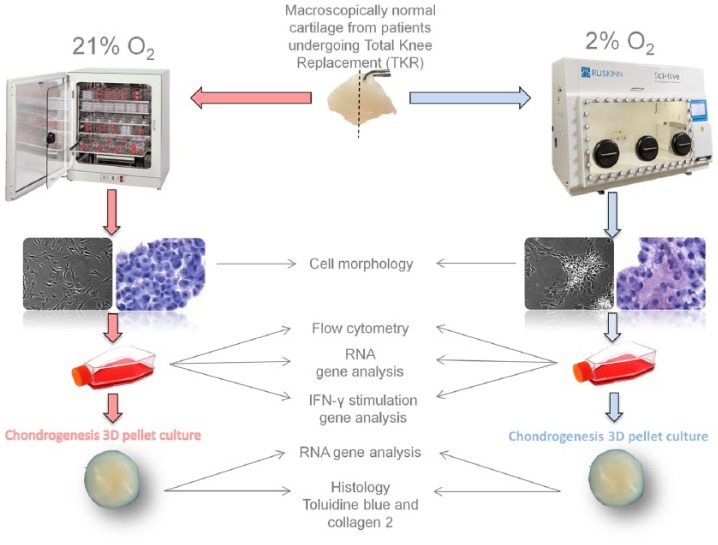
Experimental workflow. Macroscopically normal human cartilage was collected in media conditioned to 2% O_2_.

All samples were obtained after patients had provided written informed consent; favorable ethical approval was given by the National Research Ethics Service (11/NW/0875). Human cartilage was collected and processed within 3 hours of removal from 5 patients undergoing total knee replacement (TKR; **Table 1**). **Figure 1** shows an overview of the workflow. Cartilage was harvested from the condyles by the operating surgeon into DMEM-F12 medium previously conditioned to 2% O_2_ (using the HypoxyCOOL system, Baker Ruskinn Technologies Ltd). This was transferred to the SCI-tive hypoxic workstation (Ruskinn Technologies Ltd) maintained at 2% O_2_. Cartilage was then dissected and weighed in the workstation (2% O_2_), split into 2 portions and then digested overnight with collagenase II (Worthington, NJ) (CLS 2, 150-350 IU/mg dry weight) as previously described^18^ in both normoxic (21% O_2_) and hypoxic (2% O_2_) conditions. Following the enzymatic release, each tissue digest was passed through a 70-µm cell strainer (BD Biosciences) in normoxia and hypoxia; cells were recovered by centrifugation at 750 g for 10 minutes to form a cell pellet. Resulting cells were seeded at a density of 5000/cm^2^ and cultured in DMEM-F12 containing 10% fetal calf serum (FCS), ascorbic acid (50 mg/mL), and penicillin/streptomycin (P/S) and incubated at 37°C in 21% and 2% O_2_. Media conditioned to 2% was used for hypoxic cell culture throughout. A portion of NC was not put into cell culture but retained for CD immunoprofiling as a freshly isolated cell fraction (FC).

**Table 1.** Patient Demographics.

Patient	Age (Years)	Sex	Procedure	Notes	Use in Experiments
1	72	Male	TKR	Signs of OA Bone-on-bone arthritis	GK, F, MG, IG, CG
2	76	Female	TKR	Indications of OA	GK, F, MG, IG, CG
3	70	Female	TKR	Lateral meniscal tear Early signs of OA Previous microfracture that failed to heal Partial medial meniscectomy, arthroscopy, and debridement Long-term complication with postoperative pain	GK, F, MG, IG, CG
4	77	Female	TKR	Severe degenerative OA Torn medial ligament	GK, F, MG, IG
Additional patients used					
5	72	Female	TKR	Severe arthritic changes, subchondral sclerosis, large osteophyte formation in the medial compartment with some destruction of the bone.	F

GK = growth kinetics; F = flow cytometry; MG = monolayer gene expression; IG = inflammatory gene expression; CG = gene expression following chondrogenic differentiation; TKR, total knee replacement; OA, osteoarthritis.

### Calculation of Doubling Time

To calculate doubling time (DT), cells were harvested, counted, and replated when they reached 70% confluency. DT was calculated using the formula DT = (*t*_2_ − *t*_1_)ln (2)/ln (*n*_2_/*n*_1_) where *n*_2_ is the cell number at harvesting, *n*_1_ is the cell number at plating, *t*_2_ is the time at cell harvest and *t*_1_ is the time at plating.

### Centrifuge Preparation of Cells (Cytospin)

Cytospins were prepared from NC, HC (at P3-4) and FC. Cells (suspended at a density of 5 × 10^5^ cells/mL) were centrifuged at 27 × *g* for 3 minutes in a cytocentrifuge (Shandon Cytospin 2, Shandon, Runcorn, UK) to transfer the cells to dry glass slides (50 μL per slide, Cell Path Ltd, Newtown, UK). After centrifugation, the slides were air dried and stored at −20°C until use.

### Toluidine Blue Staining

Slides with cytospins or pellet sections were removed from −20°C storage and allowed to reach room temperature. They were then flooded with 1% aqueous toluidine blue (BDH, UK) stain solution for 30 seconds and rinsed in tap water. Slides were left to air dry before mounting under glass coverslips with Pertex mounting medium (Cell Path Ltd).^19^

### Immunoprofiling

Flow cytometry was used to assess the immunoprofile of chondrocytes after initial isolation (FC) and at passage 3-4 (NC and HC). Cells were harvested, filtered through a 70-μm mesh cell strainer, pelleted, resuspended in 2% bovine serum albumin (BSA) in phosphate-buffered saline (PBS) and counted. A total of 50,000 cells were used for each antibody and IgG control. A range of cell markers were used to characterize chondrocytes (16 in total), which are indicative of (1) defining MSCs according to the ISCT criteria (CD73, CD90, and CD105 positive and CD14, CD19, CD34, CD45, and HLA-DR negative); (2) chondrogenic potency (CD39, CD44, CD49c, CD151, CD166, CD271, and ROR2); (3) immunomodulation (vascular cell adhesion molecule [VCAM-1] or CD106). The appropriate isotype controls were used throughout. Cells were analyzed on a FACSCanto II flow cytometer using Diva 7 software (Becton Dickinson & Company, Oxford, UK).

### Stimulation of Cells With Interferon-γ

The pro-inflammatory cytokine, interferon-γ (IFN-γ) (Promokine, Heidelberg, Germany), was used to stimulate cells at a concentration of 25 ng/mL.^20^ IFN-γ was added to the growth media of monolayer NC and HC at 37°C for 24 hours, after which time RNA was extracted (as described below) and the expression of *IDO* assessed via reverse transcription quantitative polymerase chain reaction (RT-qPCR).

### Chondrogenic Differentiation

Pellet cultures were used to assess the chondrogenic differentiation potential of NC and HC. Cells were harvested at P3-4 in both normoxia and hypoxia. Cells (200,000 cells per pellet) were centrifuged in 1.5 mL eppendorfs (500 × *g* for 5 minutes) in 1 mL of chondrogenic medium consisting of DMEM-F12, FCS (10%), P/S (1%), insulin transferrin selenium (ITS) (1%), ascorbic acid (0.1 mM), dexamethasone (10 nM), transforming growth factor–β1 (TGF-β1) (10 ng/mL), sodium pyruvate (1 mM), and linoleic acid (20 μM). Cells were cultured for 28 days and media changed every 2 to 3 days.

### Sectioning of Chondrogenic Pellets

After 28 days, cell pellets were snap frozen in liquid nitrogen and stored at −80°C prior to use. Pellets were sectioned (7 µM) on a cryostat (Bright Instrument Co Ltd, Huntingdon, UK) onto poly-l-lysine coated slides and stained for glycosaminoglycans (GAGs) with toluidine blue metachromatic stain.

### Immunohistochemical Staining of Chondrogenic Pellets

#### Collagen Type II Staining

For collagen type II staining, sections were pretreated with 0.1% (w/v) hyaluronidase and 0.2% (w/v) trypsin (Sigma-Aldrich, UK) for 1 hour at 37°C. Sections were then washed in PBS and incubated for 2 hours at room temperature in a humidified chamber with 10 µg/mL of primary mouse monoclonal collagen type II antibody (clone CIIC1, Developmental Studies Hybridoma Bank, University of Iowa, IA, USA) in PBS. Negative control sections were incubated with a nonspecific, isotype-matched antibody (IgG1; Dako, Denmark) in place of the primary antibody at the same concentration. After incubation with the primary antibody, sections were washed in PBS before incubation with the secondary biotinylated antibody at 50 µg/mL (VECTASTAIN ABC system, Vector Laboratories Ltd, UK) according to the manufacturer’s protocol for 1 hour. To eliminate endogenous peroxidase activity sections were blocked with 0.3% (v/v) hydrogen peroxide in methanol (BDH) for 30 minutes. Labeling was enhanced with incubation of streptavidin-peroxidase for 30 minutes according to the manufacturer’s instructions (Vectastain Elite ABC kit, Vector Laboratories). Collagen type II immunopositivity was visualized by testing for bound peroxidase, detected by incubation with a substrate of diaminobenzidine tetrahydrochloride, activated by hydrogen peroxide. The sections were then dehydrated before mounting under glass coverslips with Pertex mounting medium.

#### CD49c Staining

Frozen sections were pretreated with 0.3% hydrogen peroxide (v/v) (BDH) in PBS for 10 minutes to block endogenous peroxidase. Sections were then washed 3 times in PBS and incubated for 1 hour at room temperature in a humidified chamber with blocking buffer (10% horse serum in PBS). Slides were washed 3 times in PBS before adding the primary antibody, CD49c (clone C3 II.1, at 1:250, Becton Dickinson & Company, Oxford, UK) in blocking buffer for 1 hour at room temperature.

Negative control sections were incubated with a nonspecific, isotype-matched antibody (IgG1; Dako, Denmark) in place of the primary antibody at the same concentration. Following incubation with the primary antibody, sections were washed 3 times in PBS before incubation with the secondary biotinylated antibody at 50 µg/mL (VECTASTAIN ABC system, Vector Laboratories Ltd, UK) according to the manufacturer’s protocol for 30 minutes. Labeling was enhanced with streptavidin-peroxidase (Vectastain Elite ABC kit, Vector Laboratories, Peterborough, UK) and visualized with diaminobenzadine as above. The sections were then dehydrated before mounting under glass coverslips with Pertex mounting medium.

### Measurement of 3-Dimensional Pellet Area

Chondrogenic pellets grown in normoxia and hypoxia were sectioned and stained with toluidine blue metachromatic stain (as described previously). The 3 largest sections were chosen from the middle of each chondrogenic pellet for area measurement, which was performed using NIS-Elements BR 3.2 software (Nikon, UK). This was done on 3 replicate pellet sections from each chondrogenic pellet. These were assessed from 3 different patients grown in both normoxia and hypoxia.

### RNA Isolation and RT-qPCR

RNA was extracted from the following cells: (1) monolayer NC and HC cells at P3-4, (2) chondrogenic pellets from NC and HC, (3) monolayer NC and HC cells before and after exposure to IFN-**γ**. RNA was extracted using the RNeasy Mini kit (Qiagen, Sussex, UK), following the manufacturer’s instructions. RNA was eluted from the spin column with RNAse-free water and stored at −80°C until needed. RT-qPCR analysis was performed using a SYBR green mastermix (Applied Biosystems, Warrington, UK) with hypoxanthine phosphoribosyltransferase 1 (*HPRT1*) as a reference gene (Qiagen, QuantiTect Primer Assay).

RT-qPCR was used to assess the expression of *SOX9*, aggrecan (*ACAN*) (pellet cultures only), frizzled related protein (*FRZB*), fibroblastic growth factor receptor 3 (*FGFR3*) and collagen II (*COL2A1*), which are markers indicative of chondrogenic potency. The expression of activin receptor like kinase-1 (*ALK-1*) receptor was also assessed, as its expression is associated with loss of cartilage formation.^21^ The immunomodulatory capacity of NC and HC was also assessed after stimulation with IFN-γ after 24 hours. RNA was extracted (in triplicate) from cells with and without IFN-γ stimulation. The expression of the immunomodulatory molecule, *IDO*, was measured via RT-qPCR. The reaction was conducted in the ABI 7500 RT-qPCR system (Applied Biosystems) and *C_t_* values determined using the SDS software (Applied Biosystems). Following normalization to the reference gene *HPRT1*, the presence of genes of interest (mRNA) in cells grown in hypoxia was expressed as a ratio compared with those grown in normoxia. For stimulated cells, the gene expression profile of IFN-γ-treated cells was expressed as a ratio compared to un-stimulated cells, using the comparative threshold method.^22^ A 2-fold change (up- or downregulated) was deemed biologically significant.

### Statistical Analysis

GraphPad Prism version 6 (GraphPad Software, San Diego, CA, USA) was used for statistical analysis. Data are presented as mean ± standard deviation (SD) in the graphs and text. A 2-way analysis of variance (ANOVA) with a multiple comparisons test was used to analyze flow cytometry, growth kinetics and pellet area. Levels of significance are indicated as *P < 0.05, **P < 0.01, and ***P < 0.001.

## Results

### Growth and Cell Morphology in Normoxia and Hypoxia

**Figure 2.**
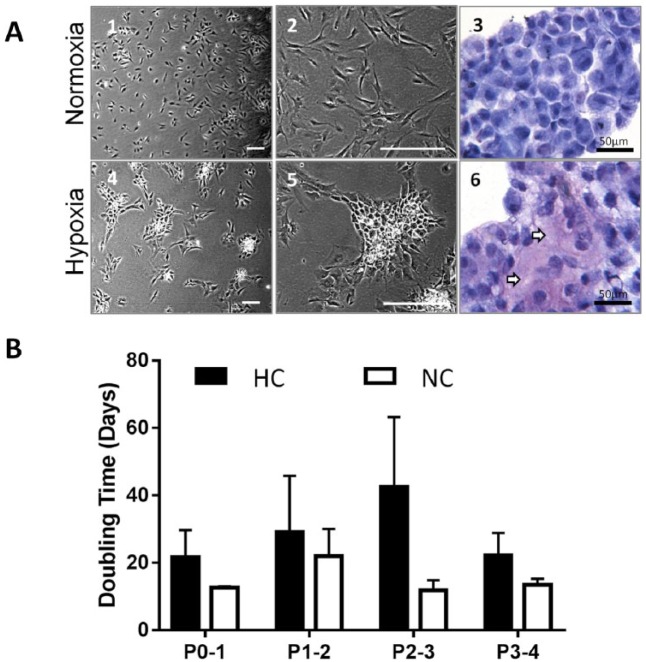
(**A**) Representative images from cells at passage 3 (P3) in normoxia (NC) (21% O_2_) and hypoxia (HC) (2% O_2_). (1 and 2) NC. (3) Cytospin stained with toluidine blue. (4 and 5) HC. (6) Cytospin stained with toluidine blue (arrows indicate glycosaminoglycan staining, purple metachromasia). (B) Doubling time (days) of NC (*n* = 4) and HC (*n* = 4). Bars represent the mean ± SD. Scale bars represent 200 µm unless otherwise stated.

**Figure 3.**
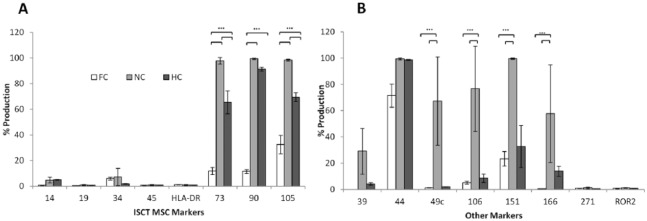
Flow cytometry data showing the presence of cell markers indicative of (**A**) mesenchymal stem cell (MSC; using the ISCT criteria). (**B**) Other markers indicative of chondrogenic dedifferentiation, potency, and immunomodulation on freshly isolated chondrocytes (FC) (*n* = 5), normoxic chondrocytes (NC) at P3-4 (*n* = 5), and hypoxic chondrocytes (HC) at P3-4 (*n* = 4). Error bars indicate the mean ± SD.

**Figure 2A** shows representative cell morphologies of NC and HC. NC appeared evenly distributed on the tissue culture plastic whereas HC grew in small clumps. This was observed for all patients through all passages and became noticeable 2 to 3 days after seeding cells. Cell cytospins stained with toluidine blue showed more purple (metachromatic) staining in HC samples compared with NC, indicative of higher GAG production (**Fig. 2A**, 3 and 6).

HC had a slower rate of growth in culture compared to NC; the mean DT of NC at P0-1 was 12.5 ± 0.7 days compared with 21.6 ± 13.9 days for HC. The greatest difference between the 2 conditions was seen at P2-3 with NC having a DT of 11.9 ± 2.9 and 42.4 ± 20.7 days for HC. However, there was no statistical difference between these conditions possibly due to a low sample number (*n* = 4 for NC and *n* = 4 for HC).

### Cell Surface Markers

Flow cytometry demonstrated that FC produced low levels of the ISCT MSC markers CD73, CD90, and CD105 (11.8%, 11.5%, and 32.4%, respectively, **Fig. 3A**) and other markers (CD44, CD49c, CD151, and CD166), which may be indicative of chondrogenic dedifferentiation when present in higher amounts (**Fig. 3B**). NC at P3-4 were positive (over ~97%) for ISCT MSC markers CD73, CD90, and CD105 and compared with FC and HC, showed increased production of CD49c, CD151, (*P* < 0.0001) and CD166 (*P* < 0.0001 and *P* < 0.001, respectively). At P3-4, HC did not fit the cell surface marker profile of MSCs as they were less than 90% positive for CD73 and CD105, this lower production was found to be significantly different to NC (*P* = 0.019 and *P* = 0.042, respectively). A significant difference was also seen for these markers on HC and FC, with FC producing the lowest levels of CD73, CD90, and CD105 (*P* < 0.0001). HC also showed significantly (*P* < 0.0001) reduced levels of CD49c, CD151, and CD166 when compared with NC. There was no significant difference for CD39 and CD271 between any of the cells tested. ROR2 was not found on any of the cells tested. These results suggest that the chondrocytes cultured in normoxia had dedifferentiated in comparison with FC populations and those cultured in 2% O_2_.

The immunomodulatory marker, CD106, was produced at very low levels on FC (mean positivity of 5.2% ± 1.07%) (**Fig. 3B**) and HC (8.1% ± 1.9%) but produced on NC at a significantly higher level (3 out of 5 NC samples analyzed were >90% positive, *P* < 0.0001).

### Chondrogenic Gene Expression in Monolayer Cells

**Figure 4.**
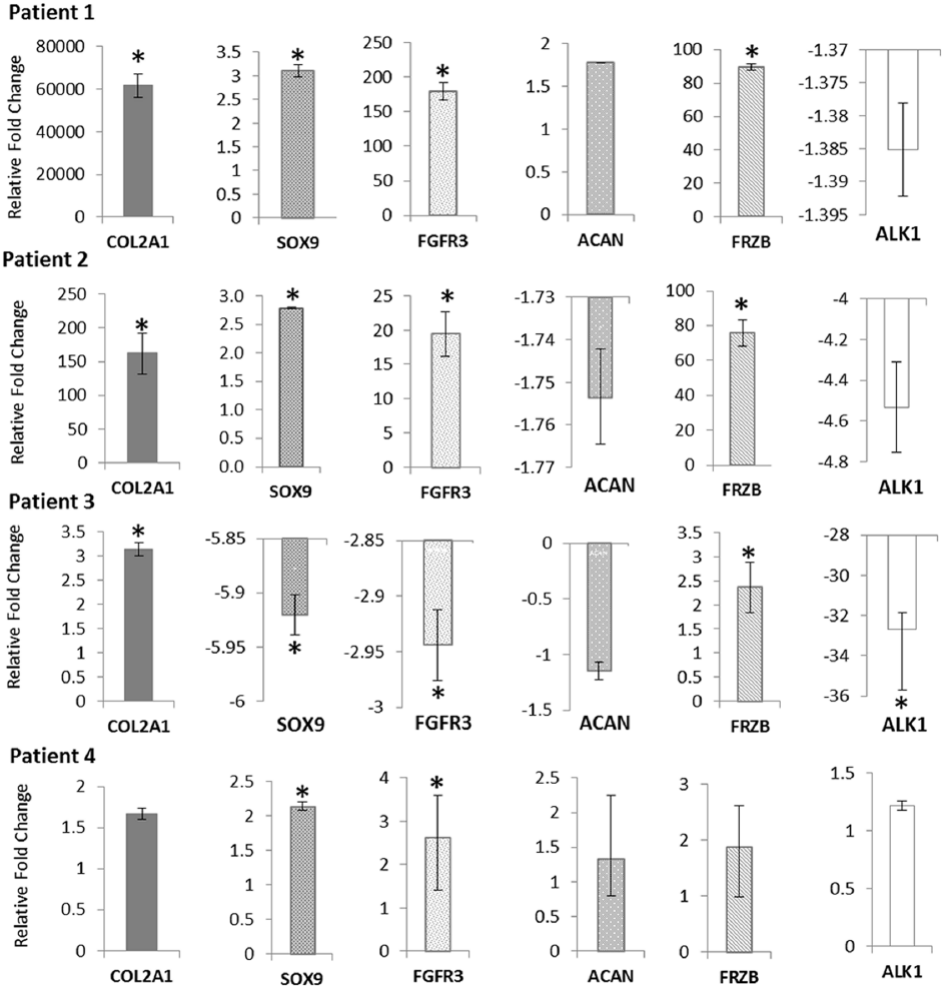
Reverse transcription quantitative polymerase chain reaction (RT-qPCR) showing the expression of genes associated with chondrogenic potency, from monolayer chondrocytes cultured in normoxia (NC) and hypoxia (HC) at passage 3-4. Gene expression for HC and NC were normalized to the reference gene *HPRT1*. Data for HC are expressed relative to NC. Stars indicate genes that are significantly up- or downregulated.

RT-qPCR analysis of several chondrogenic genes (*FRZB* and *COL2A1*) showed they were elevated in HC cultured in monolayer at P3-4 compared with NC but with high patient-patient variability (**Fig. 4**). *COL2A1* was most highly upregulated in patients 1 and 2 with 61692-fold and 163-fold higher expression in HC relative to NC, respectively. Patient 3 had a 3-fold higher *COL2A1* in HC relative to NC while patient 4 did not show any significant change. Expression of *ACAN* was not significantly altered in any patient sample following monolayer culture. *ALK1* (which has been associated with loss of cartilage formation) was not significantly up or down regulated in HC patients 1 and 4, but significantly downregulated in patients 2 and 3.

### Chondrogenic Gene Expression in Cell Pellets

**Figure 5.**
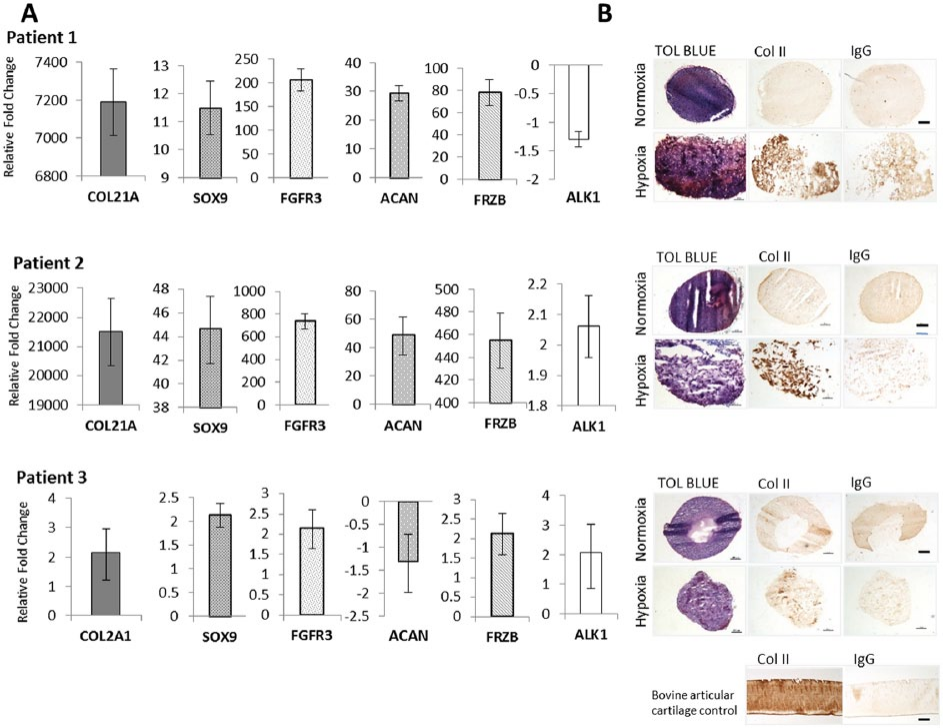
(A) Reverse transcription quantitative polymerase chain reaction (RT qPCR) showing the expression of genes associated with chondrogenic potency, from 3-dimensional chondrogenic pellets cultured in normoxia (NC) and hypoxia (HC) at passage 3-4. Gene expression for HC and NC were normalized to the reference gene *HPRT1*. Data for HC are expressed relative to NC. (**B**) Chondrogenesis was assessed after 28 days in pellet culture by staining sections for glycosaminoglycan (GAG) using toluidine blue and type II collagen by immunohistochemistry. Stars indicate genes that are significantly up- or downregulated. Scale bars represent 100 µm.

RT-qPCR analysis of the RNA from chondrogenic pellet culture was performed on only 3 patients and all showed a similar trend in gene expression to cells grown in monolayer for each patient (**Fig. 5A**). Hence, patients 1 and 2 showed the highest *COL2A1* expression with a 7190- and 21509-fold increase, respectively. *FRZB* and *SOX9* were also significantly upregulated in these 2 patients along with *ACAN*, which was expected to be elevated in 3-dimensional (3D) culture. Patient 3 showed a significant 2.14-fold upregulation of *COL2A1*. None of the other genes were significantly altered for patient 3.

### Immunohistochemical Staining of Chondrogenic Pellets with collagen II

Patients 1 and 2 showed the strongest collagen II staining in chondrogenically differentiated pellets grown in hypoxia but not in normoxia (**Fig. 5B**). This is in agreement with PCR data showing higher expression of *COL2A1* in these patients. Patient 3 showed the least staining of those grown in hypoxia but showed some small areas of collagen II staining which, again, matched the PCR data. None of the chondrogenic pellets cultured at 21% O_2_ showed positive collagen II staining from any of the patients tested.

### Measurement of Pellet Area

**Figure 6.**
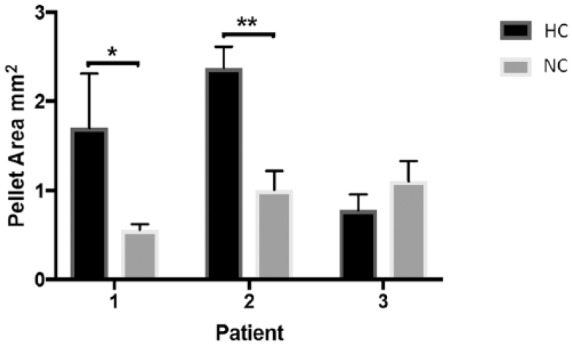
The mean area of chondrogenic pellets grown in normoxia (NC) (21% O_2_) and hypoxia (HC) (2% O_2_). Data are taken from triplicate pellets from 3 different patients. Bars indicate the mean ± SD.

Pellet area was found to be significantly larger in chondrogenic pellets grown in hypoxia compared with normoxia for patients 1 and 2 (*P* = 0.01 and *P* = 0.004, respectively), but not patient 3 (**Fig. 6**).

### Stimulation of Cells with IFN-γ and Expression of Immunomodulatory Markers

**Figure 7.**
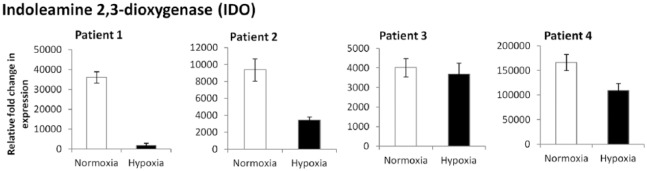
Reverse transcription quantitative polymerase chain reaction (RT-qPCR) showing the expression of the IDO gene in P3-4 monolayer chondrocytes cultured in normoxia (NC) and hypoxia (HC) following stimulation with 25 ng/mL interferon-γ (IFN-γ) for 24 hours. Gene expression was normalized to *HPRT1*. Gene expression for IFN-γ stimulated chondrocytes is expressed relative to those grown in normal media without inflammatory stimulus.

All patients showed a significant upregulation of *IDO* in NC and HC compared with baseline levels with no exposure to IFN-γ (**Fig. 7**). The expression of *IDO* in NC following stimulation with IFN-γ was higher than the expression seen in HC in patients 1, 2, and 4. Patient 3 did not show a significant difference in the expression of the *IDO* gene between NC or HC.

### Immunohistochemical Staining of Chondrogenic Pellets with CD49c

**Figure 8.**
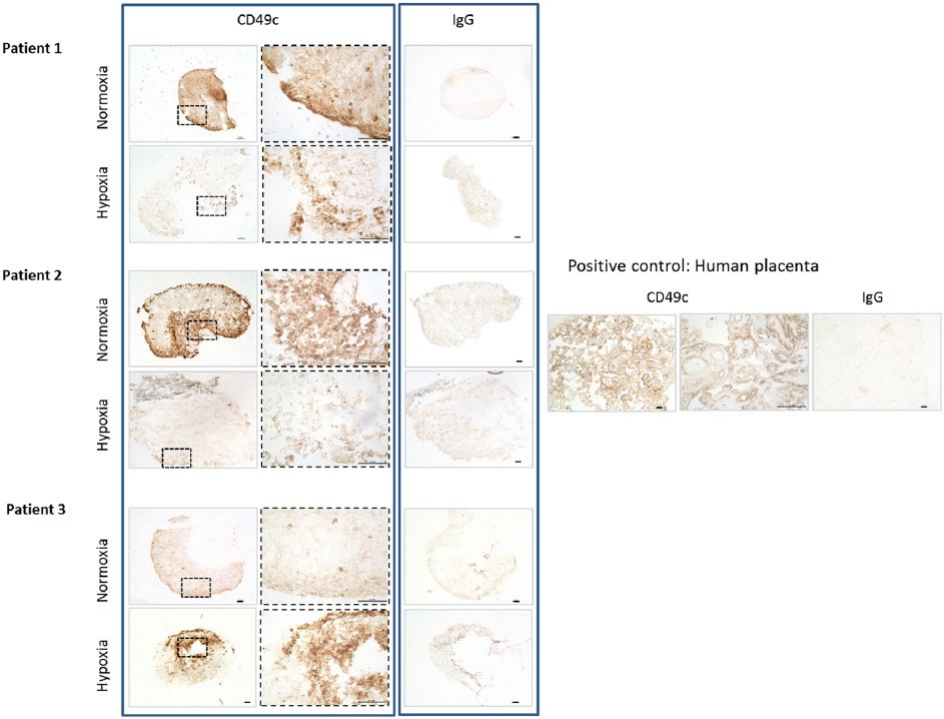
Immunohistochemistry showing CD49c staining of chondrogenic pellets cultured in normoxia (NC) and hypoxia (HC). Scale bars represent 100 μm.

Chondrogenic pellet sections from pellets cultured in normoxia stained positively for CD49c for patients 1 and 2 (**Fig. 8**), while those cultured in hypoxia for the same 2 patients showed very little positive staining. Patient 3 showed the opposite result, with hypoxic pellet sections showing the strongest staining compared with those cultured in normoxia.

## Discussion

Autologous chondrocyte implantation (ACI) has been used for more than 20 years for the treatment of cartilage injury and osteoarthritis^23^ and there is currently great interest in the advancement of this technique along with other cell therapies for the treatment of chondral and osteochondral defects. Following chondrocyte implantation into a cartilage defect *in vivo*, the level of the implanted cells contribution to the repair tissue remains largely unknown.^24,25^ The degree of dedifferentiation chondrocytes experience during normal *in vitro* cell expansion in normoxia and how this affects their therapeutic benefit when reintroduced into a diseased joint is also currently undetermined.

Therefore, the purpose of this study was to characterize and compare human articular chondrocytes, both freshly isolated and culture expanded, in normoxic and continuous noninterrupted hypoxic conditions with a workstation and equilibrated media. We hypothesized that the isolation and culture expansion of chondrocytes in continuous hypoxic conditions would slow the process of dedifferentiation and promote the upregulation of key chondrogenic genes compared with those grown in normoxic conditions.

The representative cell morphology and toluidine blue staining of NC and HC cytospins (**Fig. 2**) shows that HC grew in small clusters, compared with NC. The increased GAG production (purple metachromasia) seen in cell cytospins prepared from HC compared with NC could account for the “clumpier” cells observed in HC as GAG is known to be “sticky.” This is also in agreement with other studies showing an increase in matrix deposition in hypoxic conditions.^26,27^ As the role of chondrocytes *in vivo* at low O_2_ tensions is to maintain the cartilage matrix it was expected that HC would produce more GAG than NC.

Analysis of the doubling time of NC and HC showed that NC had a shorter mean doubling time, which was not found to be statistically significant, most probably because of the low number of samples. Higher oxygen levels (21%) encountered during *in vitro* cell expansion compared with the normal *in vivo* environment cause increased proliferation in chondrocytes, as has been shown by many other groups, due to the cells reentering the cell cycle and undergoing a shift in metabolism.^9^ Cartilage repair procedures use high cell numbers from cells expanded *in vitro*; however, there is little evidence to support the relationship between higher cell number and better cartilage repair.^28^ Therefore, the slightly slower doubling time of chondrocytes expanded in hypoxia, seen in this study, may not present a problem for standard ACI procedures.

Because of the altered cell metabolism in different oxygen tensions, differences in cell surface marker production on NC and HC were anticipated. FC, NC, and HC were all analyzed by flow cytometry using a panel of 16 cell surface markers. The reason for using MSC markers on chondrocytes (NC and HC) was due to a number of studies showing that chondrocytes revert back to a fibroblastic phenotype when grown *in vitro* in normoxia.^3,29^ As the progenitor cell for a chondrocyte is thought to be an MSC^30^ it is likely that chondrocytes become more MSC-like under these conditions. Although many markers have been studied on the “dedifferentiating” chondrocyte *in vitro*, there are no studies to our knowledge that have tested the surface marker profile after controlled and continuous hypoxic culture. Many studies assessing the effect of low O_2_ levels use intermittent hypoxia, exposing the cells to normoxic levels of oxygen during routine feeding or passaging of cells^14^ and even very short exposure to changes in oxygen tension have been shown to have profound effects on the gene expression of the chondrocytes.^31^ Gibson *et al*.^32^ showed that chondrocytes had a rapid change in metabolism, observed within 10 minutes, on reintroduction to hypoxic conditions, further highlighting the importance of the maintenance of continued hypoxia when feeding and passaging cells.

FC chondrocytes produced the lowest levels of the MSC markers CD73, CD90, and CD105 (**Fig. 3A**), while NC showed the highest percentage production. Using the ISCT criteria for cell surface marker expression, these NC would be considered to be MSCs by definition. HC produced significantly more MSC markers than FC but significantly less than NC, indicating that HC had indeed dedifferentiated but to a lesser extent than NC. Other markers, which may be indicative of chondrogenic dedifferentiation (CD44, CD49c, CD151, CD166) (**Fig. 3B**) were all produced to a very low level on FC (with the exception of CD44, which was high in all conditions), while NC showed the highest production. The markers CD166 and CD151 have both been linked to dedifferentiation of human articular chondrocytes *in vitro* in normoxic conditions and a loss of chondrogenic potency.^9,33^ The lower production by HC indicates that growth in hypoxia helped retain chondrogenic potency compared with those grown in normoxia. Previous work in our group compared the cell surface marker profile of chondrocytes and MSCs (isolated and maintained at 21% O_2_) and found no significant difference between the ISCT MSC markers or for CD49c and CD166. However, CD106 was not tested in this previous work.^34^

The immunomodulatory marker, vascular cell adhesion molecule 1 (VCAM-1) or CD106 has been associated with a distinct subpopulation of MSCs with unique immunosuppressive activity.^35^ Its classical function is thought to be through mediation of the adhesion of lymphocytes and monocytes to vascular endothelium.^36^ The higher production of CD106 on NC compared with HC and FC highlights a further stronger similarity of NC to MSCs, than native chondrocytes. A limitation of using freshly isolated chondrocytes, for comparison in flow cytometry, is that the enzymes used to break down the cartilage matrix and free the cells could cleave cell surface antigens, making them appear to be produced at a lower level. One particular study^9^ showed that chondrocytes reexposed to collagenase overnight (following initial tissue digestion) had the same level of cell surface marker expression as those without collagenase exposure, however overnight incubation with pronase (not used in our study) caused reduced cell surface marker detection via flow cytometry on 9 of the 11 markers tested. This would indicate that the results seen in our study are likely to be a true representation of the production of cell surface markers on freshly isolated chondrocytes.

Reduced oxygen levels are associated with induction of elevated expression of key chondrogenic genes such as *COL2A1, SOX9*, and *ACAN*,^5,37^ which is in agreement with the results seen in this study. The re-direction toward proliferation in normoxic conditions is also associated with reduction in endogenous collagen type II and proteoglycan production^9^ seen in this study, as none of the NC differentiated in 3D pellet culture showed any positive collagen II immunostaining.

**Table 1** shows the patient demographics for samples used in this study. Patients 1 and 2 had early osteoarthritic changes, which may explain why their chondrocytes were still able to upregulate genes associated with chondrogenic potency and showed positive collagen II immunostaining in 3D pellet culture in hypoxia. Patient 3, although having early signs of OA, had a previous microfracture, which had failed to heal, a meniscal tear leading to a meniscectomy and long-term complications with postoperative pain. Patient 4 had severe degenerative OA as well as a torn medial ligament. Therefore, it is likely that chondrocytes isolated from patients 3 and 4 may have lost the ability to upregulate genes associated with chondropotency and therefore the ability to produce cartilage specific ECM and form stable cartilage *in vivo*. The lack of collagen II immunostaining seen in 3D chondrogenic pellets in patient 3 supports the loss of *COL2A1* as a potency gene seen in the PCR data. However, more patients with severe OA versus early OA would need to be assessed in this way.

Because of the significant differences in the production of CD49c on monolayer cells cultured in normoxia and hypoxia, chondrogenically differentiated pellets were also assessed for production of this cell surface marker, via immunohistochemistry. CD49c (integrin α-3) is a protein encoded by the *ITGA3* gene. The alpha chain integrin family function as cell surface adhesion molecules, joining with a beta 1 subunit forming an integrin that interacts with extracellular matrix proteins.

Results revealed variable staining between the 3 patients tested. The positive CD49c staining in chondrogenic pellets from patients 1 and 2 (**Fig. 8**) cultured in normoxia and lack of staining in matched pellets from hypoxia, taken together with the PCR data showing that hypoxic culture retained the best chondrogenic phenotype, may indicate that CD49c is a marker for dedifferentiation. This is in agreement with other studies assessing changes in the cell surface marker profile on chondrocytes and MSCs in normoxia. CD49c has been shown to increase on chondrocytes cultured in normoxia over several passages^38^ and to become down regulated on human MSCs on chondrogenic differentiation.^39,40^ However none of these studies assessed human chondrocytes under hypoxic conditions. Conversely, chondrogenic pellets from patient 3 cultured in hypoxia showed stronger positive staining for CD49c compared with those cultured in normoxia. As discussed earlier, this patient’s cells may have already dedifferentiated, which may explain the positive staining for CD49c in hypoxic pellets; however, this does not explain the comparative weaker staining seen in normoxic chondrogenic pellets for the same patient. More patients would need to be assessed in further work to gain an understanding of the relationship between CD49c and dedifferentiation. CD49c warrants further investigation as a possible marker for chondrogenic dedifferentiation.

Chondrogenic pellets grown in hypoxia from patients 1 and 2 (**Fig. 6**) had significantly larger pellets than those grown in normoxia (*P* = 0.01 and *P* = 0.004, respectively). In accordance with our study, Babur *et al*.^41^ found larger chondrogenic pellet sizes after culture in hypoxia (in this case 3% O_2_) compared with normoxia (21% O_2_). The difference in pellet size was thought to be due to the higher GAG content in hypoxic pellets. Interestingly, these cells were also found to be more metabolically active than those cultured in normoxia. The lack of any size difference and positive collagen II staining for the cell pellets from patient 3 could indicate that they had already dedifferentiated.

Previous work in our group has shown that MSCs highly upregulate IDO following IFN-γ stimulation^42^ and production of IDO is classically associated with MSCs not chondrocytes; however, the higher gene expression seen in NC in this study could be explained by a higher degree of de-differentiation toward an MSC lineage. Lohan *et al*.^43^ showed that culture expanded primary chondrocytes grown in 19% O_2_ and 2% O_2_ have potent immunomodulatory properties, although no studies have shown the production of IDO in chondrocytes in normoxic and hypoxic conditions. The higher upregulation of *IDO* in NC from patients 1, 2, and 4 could indicate a higher degree of dedifferentiation toward an MSC phenotype. As there was no difference in expression in NC and HC from patient 3, together with a lack of chondrogenic marker expression, it is likely that this patient’s cells had already dedifferentiated.

## Conclusion

These results suggest that chondrocytes, like MSCs, are capable of immunomodulatory behavior and may be able to dampen down an immune response and calm inflammation *in vivo*; these properties are likely to be enhanced by culture expansion in normoxia. Immunomodulatory molecule expression and cell surface markers indicative of a MSC phenotype were reduced in hypoxia, which may indicate the maintenance of a chondrogenic phenotype when cultured at 2% O_2_. This is supported by upregulation of key genes associated with chondrogenesis in hypoxia, which confirms the findings of other studies. Hence, the mechanism of action of chondrocytes culture expanded in 21% O_2_ for cartilage repair may be more immunomodulatory, rather than formation of the repair cartilage due to a loss of chondrogenic potency.
